# The effect of intravenous granisetron on prophylactic ephedrine for preventing hypotension after general anaesthesia induction in elderly patients: a randomized controlled trial

**DOI:** 10.1038/s41598-023-38303-6

**Published:** 2023-07-18

**Authors:** Youfa Zhou, Cencen Wang, Binbin Lin, Xianyi Lin, Yanting Zhang, Xin Yu, Gang Chen

**Affiliations:** 1 Department of Anesthesiology, Sir Run Run Shaw Hospital, School of Medicine, Zhejiang University, Qingchun East Road No. 3, Hangzhou, 310020 China; 2 Department of Anesthesiology, The First People’s Hospital of Hangzhou Lin An District, Hangzhou, China

**Keywords:** Clinical pharmacology, Clinical trial design

## Abstract

Serotonin 3 receptor antagonists, a commonly used drug for preventing postoperative nausea and vomiting, have recently been reported to decrease the incidence of hypotension and the need for vasoactive drugs after spinal anaesthesia in obstetric surgery. However, it remains unknown whether they could also prevent hypotension after induction of general anaesthesia. In the current study, we aimed to investigate the effect of intravenous granisetron on prophylactic ephedrine for preventing hypotension after general anaesthesia induction in elderly patients. Sixty elderly patients were randomly assigned to receive granisetron or saline control 30 min before induction of general anaesthesia. The first patient in each group received a prophylactic dose of ephedrine (0.15 mg kg^−1^) to prevent hypotension. The prophylactic dose for each patient was increased or decreased by 0.05 mg/kg based on the efficacy results of the previous patient. The up-down sequential allocation analysis and probit regression was used to calculate the effective dose for 50% of patients (ED50) with prophylactic ephedrine. In the up-down sequential allocation analysis, the ED50 of ephedrine was significantly lower in group granisetron (0.08 mg kg^−1^ [95% CI, 0.06–0.11 mg kg^−1^]) when compared with group control (0.14 mg kg^−1^ [95% CI, 0.13–0.16 mg kg^−1^]) (P < 0.001). The conclusion was further supported by probit regression analysis (0.09 mg kg^−1^ [95% CI, 0.05–0.12 mg kg^−1^] in group granisetron and 0.14 mg kg^−1^ [95% CI, 0.12–0.16 mg kg^−1^] in group control). Intravenous granisetron reduced the requirement of prophylactic ephedrine in preventing hypotension after general anaesthesia induction in elderly patients.

## Introduction

Hypotension is a common complication in patients under general anesthesia^1^, which may lead to organ hypoperfusion and ischemia^2^. Severe episodes of intraoperative hypotension have been reported to be related to the occurrence of various postoperative adverse events^3–7^. Patients are most likely to suffer from hypotension during induction of general anesthesia due to the depression of cardiac function and vasodilation caused by anesthetic drugs, as well as lack of surgical stimulation, particularly in the elderly patients^8,9^. The elderly patients are more vulnerable to the hypotension associated postoperative adverse outcomes due to age-related changes in cardiovascular physiology^3^. Therefore, prevention of hypotension after general anesthesia induction in the elderly is of great significance.

Ephedrine is an α- and β-adrenergic agonist that increases peripheral vascular resistance, cardiac output, and heart rate and thus maintains blood pressure. Prophylactic use of ephedrine at different doses (0.03–0.2 mg/kg) has been indicated to improve hypotension response after general anesthesia to varying degrees^10–12^. However, as the dose of ephedrine increases, a minority of people may develop hypertension, tachycardia, or even arrhythmia, which may endanger the lives of patients^10,13^. Therefore, a comprehensive knowledge of the best effective ephedrine dose and a reduction in ephedrine requirement may be of great benefit.

Serotonin 3 receptor antagonists are routinely used at the end of surgery for preventing postoperative nausea and vomiting (PONV) in patients at high risk for PONV during general anesthesia. They have been reported to reduce the incidence of hypotension and the need for vasopressin in obstetric and non-obstetric patients during intraspinal anesthesia^14,15^. It was reported that prophylactic use of ondansetron can effectively prevent hypotension after induction of general anesthesia in the elderly^16^. However, whether 5-HT3 receptor antagonists would reduce the need of vasopressin for preventing hypotension after induction of general anesthesia in elderly patients remain exclusive.

In this study, we sought to determine the effective dose of prophylactic ephedrine for 50% of elderly patients who received a single intravenous dose of granisetron or placebo before induction in preventing hypotension after induction. This study will provide important reference for the effective dose of prophylactic ephedrine in preventing hypotension after general anesthesia induction and the optimal timing of granisetron administration in elderly patients at high risk for PONV.

## Materials and methods

### Study design and participants

This study was approved by the Ethics Committee of Sir Run Shaw Hospital, School of Medicine, Zhejiang University (IRB No. 20200928-30) and all patients who participated in the study signed informed consent before recruitment. The trial was registered prior to patient enrollment at Chinese Clinical Trial Registry (registration number, ChiCTR2000040344; Principal investigator: Gang Chen, date of registration, November 28, 2020), adhered to the applicable CONSORT guidelines, and complied with the Declaration of Helsinki.

From December 1, 2020 to January 31, 2021, sixty patients who were aged 60–75, American Society of Anesthesiologists (ASA) Physical Status I or II, with a body mass index (BMI) between 18.5 and 24, with a simplified risk score^17^ for predicting postoperative nausea and vomiting (PONV) greater than 1, competent to provide informed consent, and scheduled for selective non­cardiac surgeries under general anaesthesia were recruited. Exclusion criteria included: a history of severe vascular disease, unstable angina, respiratory distress, autonomic nervous system disorders, diabetes mellitus or mental incompetence. Patients with hypotension, hypertension, severe hepatic or renal insufficiency were also excluded. Written informed consent was obtained from all participants before enrollment.

Patients were randomly allocated to receive intravenous injection of granisetron (3 mg/3 ml, group G) or saline placebo (3 ml, group C) prepared in a uniform appearance syringe within 30 s half an hour before induction of general anesthesia. Randomization scheme adopting computer-generated random number codes was prepared by statisticians, and other investigators involved in subsequent study were unaware of patient sequence. Patients allocations were kept in opaque, sealed envelopes that were not opened till the participants entered the pre-operating room.

Premedication with 0.02 mg/kg intravenous midazolam was given 30 min before induction in the pre-operating room. When patients arrived the operating room, standard monitoring (electrocardiogram, SpO_2_ and automated noninvasive arterial pressure by oscillometry) was established. We defined the basal blood pressure (BP) and heart rate (HR) as the means of three consecutive BP and HR measurements at 1-min intervals. All patients were preoxygenated with oxygen 100% via a face mask for 3 min before the induction of general anesthesia. An i.v. bolus of sufentanil 0.5 μg/kg was administrated 1 min after the start of preoxygenation in 1 min. One minute after sufentanil injection, ephedrine was injected and then anaesthesia was induced with propofol 2 mg/kg. After induction of anaesthesia, cisatracurium 0.15 mg/kg was given to facilitate tracheal intubation. Mask ventilation was done for 3 min till tracheal intubation performed by an anaesthesiologist with at least 5 years of experience and blinded to group allocation by video-laryngoscopy. Oral tracheal tubes (7.0 and 7.5 mm, internal diameter) were used for female and male patients, respectively. Anaesthesia was maintained with sevoflurane 1–1.5%, propofol 4 mg/kg h, remifentanil 5 μg/kg h and dexmedetomidine 0.5 μg/kg h 5 min after intubation to maintain the BIS value between 40 and 60.

Patients’ systolic blood pressure (SBP), diastolic blood pressure (DBP), mean arterial pressure (MAP), heart rate, and oxygen saturation were recorded on arrival in operation room (T_baseline_), just before induction T1, 2 min after induction (T2), just before intubation (T3), 1 min after intubation (T4), 2 min after intubation (T5), 5 min after intubation (T6) and 10 min after intubation (T7). An intravenous bolus of ephedrine (3 mg) was administrated to treat episode of hypotension. An intravenous bolus of atropine (0.3 mg) was adopted for treatment of significant bradycardia (HR less than 45 beats/min)^10^.

Hypotension after induction of anesthesia were defined as a more than 25% decrease in MBP from the baseline level during the observation phase of the study^18^. The first patient in each group received a prophylactic dose of ephedrine (0.15 mg/kg) which was the average of the two intervention groups in a previous study^10^. We determined the prophylactic dose for each subsequent patient based on the drug responsiveness of the previous patient as previous reported^19^. If the prophylactic dose of ephedrine was effective to prevent the episode of hypotension in the period after induction of anesthesia in the previous patient, the prophylactic dose of the subsequent patient was decreased by 0.05 mg/kg. If the prophylactic dose was not effective in the previous patient, the prophylactic dose of the subsequent patient was increased by 0.05 mg/kg.

### Statistical analysis

We decided to recruit 30 patients in each group because previous studies have suggested that data from 20 to 40 patients are sufficient to reliably estimate the ED50 for most scenarios in up-and-down method^20^. The ED50 of prophylactic ephedrine was calculated by taking the average of values for the midpoints of pairs for the dose in study subjects in which the effect of prevention hypotension went from ineffective to effective (crossover). The 95% confidence interval (CI) and standard error for ED50 values were calculated as previous study reported^21^, and the difference of ED50 values was analyzed using Student *t* test. Continuous data were analyzed using Student *t* test or Mann–Whitney *U* test according to the results of test for normality by Kolmogorov–Smirnov test. Categorical data were analyzed using χ^2^ or Fisher exact tests, as appropriate. Probit regression analysis is commonly used to estimate dose–response curves and ED50 from data on the dose–response relationship of the drug, where the response variable is binary (e.g., alive or dead, cured or not cured). In the current study, we calculated the numbers of “effective” and “ineffective” patients at different dose category in the two groups and estimated the dose–response curves and ED50 by probit regression as sensitivity analysis and overlapping CI methodology was used to test the differences in ED50 values between groups^22^. The ED50 of the two groups can be considered to be statistically different as long as the confidence intervals of the two groups do not overlap more than 83%^22^. All statistics were performed by GraphPad Prism version 6.0 (GraphPad Software Inc, San Diego, CA) and IBM SPSS Statistics for Windows version 25.0 (IBM Corp, Armonk, NY). A P value of less than 0.05 (two-tailed) was considered statistically significant.

## Results

**Figure 1:**
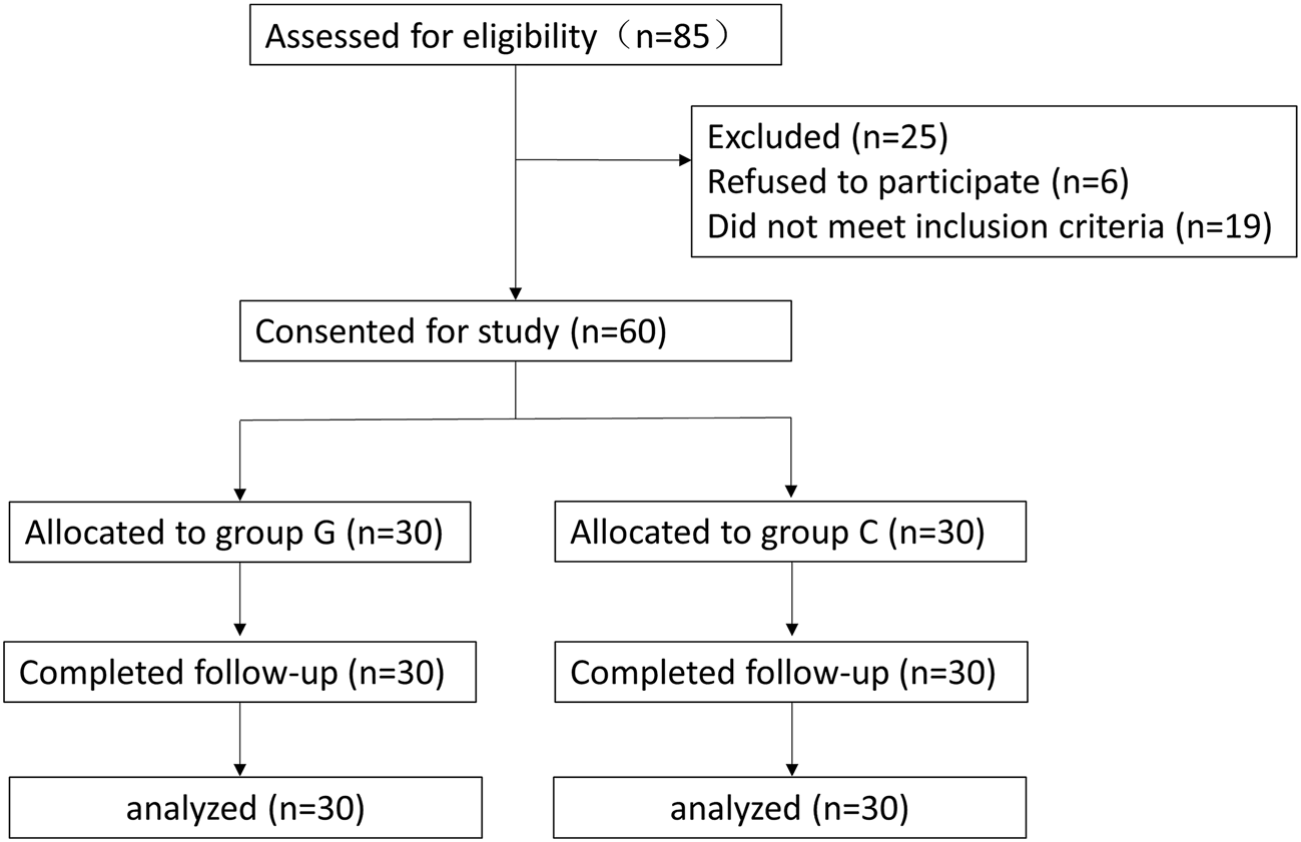
CONSORT flow diagram of patient inclusion.

Overall, 85 patients were assessed for eligibility, 19 patients did not meet inclusion criteria and 6 patients refused to participate. Finally, a total of 60 patients provided informed consent, underwent randomization, and completed the study, with 30 patients in each group. The Consolidated Standards of Reporting Trials (CONSORT) diagram for recruitment to the trial is presented in Fig. 1. Patient characteristics were similar between groups as shown in Table 1.

**Table 1:** Characteristics of the study subjects. Continual data are expressed as mean (SD); and absolute numbers for categorical data.

Characteristic	Group C (n = 30)	Group G (n = 30)	P value
Age	64.3 (3.54)	64.6 (3.82)	0.55
Sex (male/female)	12/18	15/15	0.44
Height (cm)	162.0 (8.94)	161.2 (7.71)	0.71
Weight (kg)	56.7 (8.41)	58.22 (6.83)	0.45
Simplified risk score for PONV	2.3 (0.22)	2.4 (0.15)	0.62
Body mass index (kg/m^2^)	21.5 (1.67)	21.8 (1.51)	0.47
ASA (I/II)	11/19	13/17	0.56
Baseline MBP (mmHg)	102.2 (8.53)	103.1 (8.57)	0.68
Baseline HR (beats/min)	65.4 (5.8)	67.8 (5.3)	0.10

*MAP* mean arterial pressure, *HR* heart rate, *ASA* American Society of Anesthesiologists physical status.

**Figure 2:**
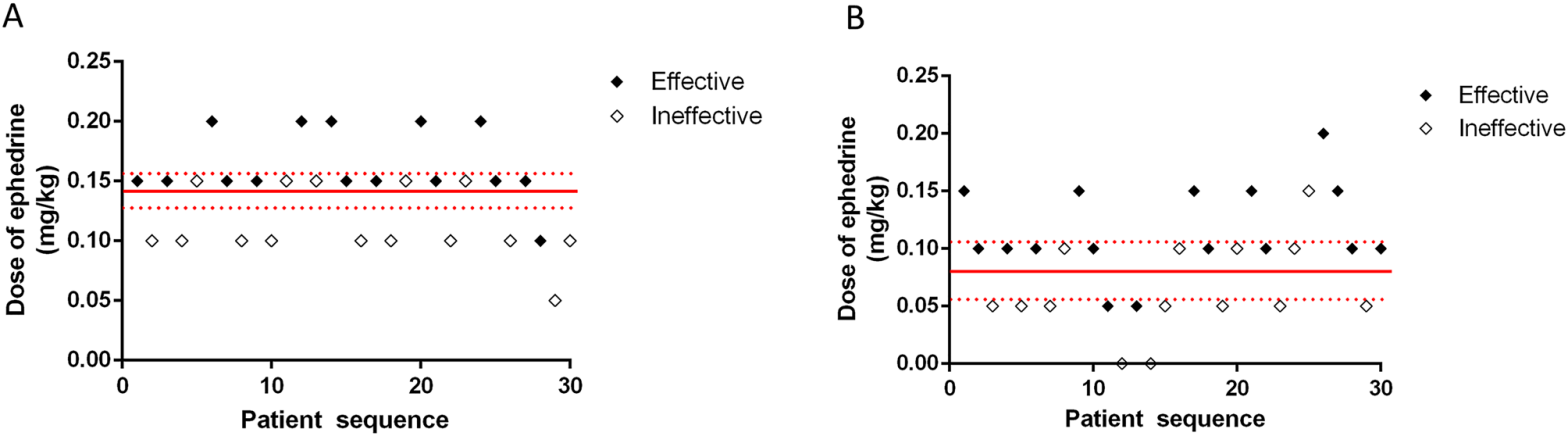
Individual response to prophylactic ephedrine at corresponding dosage in group C (**A**) and group G (**B**). The ED50 of intravenous ephedrine dosage calculated by up-down analysis was 0.14 mg/kg (95% CI, 0.13–0.16 mg/kg) in group C, and 0.08 mg/kg (95% CI, 0.06–0.11 mg/kg) in group G. Solid lines represent the ED50 values, and dashed lines represent the 95% CI. *ED50* effective dose in 50% of subjects, *CI* confidence interval.

**Figure 3:**
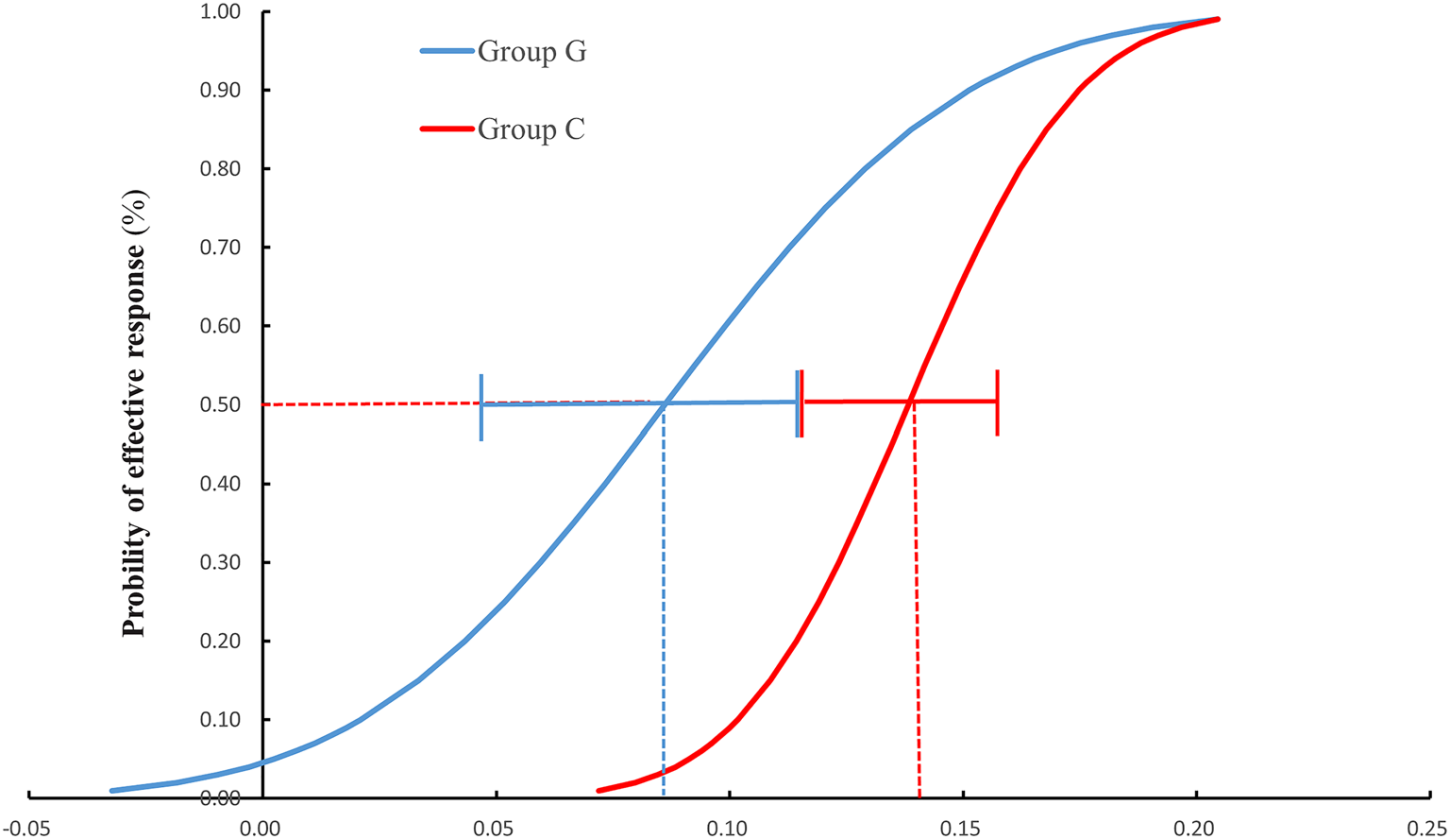
Dose–response curve and 95% confidence interval for the ED50 of ephedrine in two groups. The ED50 of intravenous ephedrine dosage calculated by probit regression was 0.09 mg/kg (95% CI, 0.05–0.12 mg/kg) in group G and 0.14 mg/kg (95% CI, 0.12–0.16 mg/kg) in group C. *ED50* effective dose in 50% of subjects, *CI* confidence interval.

The response of patients in two groups to the different prophylactic doses of ephedrine for prevention of hypotension after induction is shown in Fig. 2. The ED50 of ephedrine for the effective prevention of hypotension after induction in group C and group G was 0.08 mg/kg (95% CI, 0.06–0.11 mg/kg) and 0.14 mg/kg (95% CI, 0.13–0.16 mg/kg), respectively, which suggested that administration of granisetron before induction of anesthesia significantly reduced the ED50 of prophylactic ephedrine for prevention of hypotension during induction (P < 0.001). Dose–response curves for the prophylactic dose of ephedrine in the two groups for preventing hypotension are presented in Fig. 3. The ED50 values estimated by probit regression were 0.09 mg/kg (95% CI, 0.05–0.12 mg/kg) in group G and 0.14 mg/kg (95% CI, 0.12–0.16 mg/kg) in group C, respectively. The overlapping CI methodology also supported that ED50 of ephedrine was significantly lower in group G compared with that in group C. There was no significant difference in incidences of adverse events including hypotension, hypertension, bradycardia, and tachycardia between groups (Table 2).

**Table 2:** Incidences of adverse events. Values are number (proportion).

Adverse events	Group C (n = 30)	Group G (n = 30)	P value
Hypotension	15 (50%)	14 (46.67)	0.7961
Hypertension	1 (3.33%)	0 (0%)	0.3121
Bradycardia	1 (3.33%)	2 (6.67%)	0.5536
Tachycardia	2 (6.67)	0 (0%)	0.1503

## Discussion

The current study is the first randomized placebo-controlled study that investigates the effect of intravenous granisetron on the ED50 of ephedrine for preventing hypotension after general anesthesia induction in elderly patients. We found that the ED50 of ephedrine for preventing hypotension after general anesthesia induction in elderly patients was about 0.14 mg/kg. Moreover, we demonstrated that prophylactic IV administration of granisetron before general anesthesia induction results in a significantly lower ephedrine requirement to prevent post-induction hypotension which was supported by a significant reduction of ED 50 to 0.08 mg/kg in granisetron group.

Induction of general anesthesia often results in a significant reduction in arterial blood pressure, especially in the elderly^23^. Many mechanisms are associated with general anesthesia-induced hypotension, such as reduced systemic vascular resistance^24^ and cardiac output caused by a combination of vasodilation, decreased myocardial contractility^25^, and impaired baroreflex mechanisms^26^ induced by general anesthesia induction drugs. There is still no accepted definition standard intraoperative hypotension^27^. A common criterion is a > 30% decrease in intraoperative mean arterial pressure compared to the first measurement in the operating room, but this magnitude of hypotension significantly increases the risk of perioperative stroke in elderly patients. Thus, hypotension was defined as a decrease of more than 25% from basal blood pressure in the current study^28^.

Numerous previous studies investigated the effect of 5-HT_3_ receptor antagonists on hypotension induced by spinal anesthesia^29–31^. Interestingly, the results of these studies were inconsistent. Rashad and Farmawy found that prophylactic use of 5-HT_3_ receptor antagonists in cesarean section can significantly reduce the dose of vasoactive drugs for preventing hypotension^32^, while Behdad and colleagues elucidated that the granisetron did not influence hypotension and bradycardia induced by spinal anesthesia^33^. A meta-analysis including 17 randomized controlled trials demonstrated that prophylactic administration of 5-HT_3_ antagonists can effectively reduce the incidence of hypotension and bradycardia in the obstetric population^14^. Nevertheless, this meta-analysis did not concretely quantify the results of drug dosage. Both granisetron and ondansetron are common 5-HT receptor antagonists with the similar mechanism of action. However, as a highly selective serotonin receptor antagonist, granisetron has a low or weak affinity for other serotonin receptors, such as histaminergic and dopaminergic receptors^34^. A previous study reported that granisetron was more effective than ondansetron in attenuating the decrease of MAP, which is clinically significant^31^. Therefore, we elaborated the effects of granisetron on the hemodynamics after general anesthesia in elder patients. We explored the dose–effect relationship of ephedrine in preventing hypotension after general anesthesia induction by calculating ED50 through up and down method and demonstrated that granisetron reduced the ED50 of ephedrine in preventing hypotension after induction.

The exact mechanism of serotonin 3 receptor antagonists in reducing hypotension and the need of vasopressin is still not fully understood. A possible explanation for this phenomenon is that such drugs block the Bezold–Jarisch reflex which is associated with perioperative hypotension^35^. Bezold–Jarisch reflex could be triggered by reduced cardiac venous return associated with peripheral vascular dilatation induced by general anesthesia induction and lead to bradycardia which further aggravated hypotension^35^.

Among the receptors involved in Bezold–Jarisch reflex are chemoreceptors responding to 5-hydroxytryptamine3 (5-HT3, serotonin)^36^. Therefore, serotonin 3 receptor antagonists could theoretically prevent Bezold–Jarisch reflex based on chemoreceptors sensitive to 5-HT3. Animal studies have demonstrated the effectiveness of 5-HT3 antagonists in blocking Bezold–Jarisch reflex^37^. Moreover, 5-HT3 antagonists have been widely reported to reduce hypotension and the need of vasopressin in spinal anesthesia which may also attributed to the blockage of Bezold–Jarisch reflex^14^. Although no study has explored the probability of the Bezold–Jarisch reflex in elderly patients, previous studies have concluded that the age distribution of the Bezold–Jarisch reflex is bimodal^38^. Young patients are more likely to develop bradycardia, while elders are more likely to develop hypotension^38^. Hypotension and bradycardia are common in elderly patients following induction of general anesthesia, suggesting the potential role of 5-HT3 receptor antagonists in preventing hypotension after induction in the elderly patients.

The simplified risk score for PONV is a tool used to predict the likelihood of a patient experiencing PONV after surgery. It consists of four factors: female gender, non-smoking status, history of PONV or motion sickness, and the use of opioids for postoperative pain management. Each factor is assigned one point, and patients with a score of 2 or higher are considered to be at high risk for PONV^17^. Prophylactic use of granisetron at the end of surgery was highly recommended to prevent PONV especially in patients at high risk^39^. Therefore, our study also has important implications for the timing of prophylactic use of granisetron in aged patients at high risk for PONV.

Our current study showed no significant differences in adverse events between the two groups. There are two possible reasons for this phenomenon. One possibility is that granisetron at this dose itself has no significant effect on hypotension and hypertension. On the other hand, it could be due to our research protocol. We adopted the sequential up-down method, and the ephedrine dose of the next patient was constantly adjusted according to the blood pressure result of the previous patient, so that the incidence of hypotension and hypertension was not significantly different between the two groups.

The present study has several limitations. First, Blood pressure measurements may be affected by the size and location of the cuff and the type of monitor used. Second, the blood pressure measurement was intermittent and hypotension occurring between measurements might not have been detected. Third, the definition of intraoperative hypotension is variable, which significantly limited the comparisons between studies concerning this issue. Forth, different types of surgery included in this study may affected the reliability of this study. However, the inclusion of different types of surgery can better reflect real clinical situation. Fifth, the effect of different doses of granisetron on the prevention of hypotension after general anesthesia remains to be studied.

## Conclusions

The current study suggested that the ED50 of prophylactic ephedrine in preventing hypotension after general anesthesia induction in elderly patients at high risk for PONV in granisetron group was significantly lower compared with that in placebo group. Therefore, administration of granisetron prior to induction of anesthesia appears to be a more reasonable timing for elderly patients at high risk for PONV.

## Data Availability

The de-identified data for individual participants underlying our results can be accessed with approval from the corresponding author 6 months after publication. The study protocol, statistical analyses, and clinical study report will also be available.
